# Thermoregulatory responses to air temperature of −5 °C at different wind speeds: significance of strong wind in a mild cold environment

**DOI:** 10.1186/s40101-025-00419-1

**Published:** 2026-01-12

**Authors:** Do-Hee Kim, Kyu Rang Kim, Cho-Eun Lee, Gyeongri Kang, Heeyoung Ju, Jeong-Kyun Ju, Joo-Young Lee

**Affiliations:** 1 Research Institute for Human Ecology, Seoul National University, 1 Gwanak-Ro, Gwanak-Gu, Seoul, 08826 Republic of Korea; 2 Research Applications Department, National Institute of Meteorological Sciences, Seogwipo, Republic of Korea; 3 Department of Fashion and Textiles, Seoul National University, 1 Gwanak-Ro, Gwanak-Gu, Seoul, 08826 Republic of Korea; 4 FITI Testing & Research Institute, 79 Magokjungang 8-Ro 3-Gil, Gangseo-Gu, Seoul, 07791 Republic of Korea; 5 Graphene Research Center for Convergence Technology, Advanced Institute of Convergence Technology, 145, Gwanggyo-Ro, Yeongtong-Gu, Suwon-Si, Gyeonggi-Do, Republic of Korea

**Keywords:** Wind speed, Cold stress, Cold strain index, Core temperature, Shivering, Subjective responses, Body surface area, Morphological factors

## Abstract

**Background:**

Air temperature that is considered as cold varies according to individuals. Urban people who live in temperate climates are accustomed to mild cold with varying wind speeds, but relatively few studies have examined the effects of wind speed in mild cold on individuals wearing winter clothing, especially compared to studies conducted in severe cold environments. We examined thermoregulatory responses to varying wind speeds in mild cold, considering anthropometric characteristics of individuals.

**Methods:**

Ten healthy males (23.9 ± 3.3 years in age, 175.8 ± 4.9 cm in height, 74.4 ± 7.0 kg in body weight) participated in the following four wind conditions (0, 2, 4.5, and 7 m·s^−1^) at an air temperature of −5 °C (wind chill temperature: −5 to approximately −12 °C). Subjects wore winter clothing (*I*_T_, 2.1 clo), and every trial consisted of 80 min (10-min rest, 60-min walking, and 10-min recovery).

**Results:**

Rectal and gastrointestinal temperatures remained stable across all wind conditions, suggesting sufficient insulation from the winter clothing. However, peripheral skin temperatures decreased significantly with higher wind speeds (all *P*s < 0.05), with finger temperature averaging 12.7 °C at 7 m·s^−1^. Overweight subjects showed less frequent shivering than normal-weight subjects. Both body surface area (BSA) and body mass index (BMI) were negatively correlated with overall thermal comfort and positively correlated with shivering frequency (all *Ps* < 0.05). BSA was also negatively correlated with toe temperature (*P* = 0.001).

**Conclusions:**

While typical winter clothing (2.1 clo) effectively maintains core temperature in wind chill conditions down to −12 °C, extremities, particularly the hands, require better insulation. Peripheral skin temperatures and thermal comfort provide reliable indicators for assessing cold stress. Morphological properties of the body also influenced cold responses, with overweight individuals exhibiting less frequent shivering and larger body surface areas correlating with greater cold sensitivity. These findings offer insights into optimizing winter clothing design to improve comfort and safety in windy conditions in mild cold.

## Background

There is no universally accepted definition of “cold,” but temperatures below 0 °C are generally considered cold [1]. Recent years have witnessed record-breaking extreme temperature events and cold waves in the USA and East Asian countries, highlighting the need to investigate the impacts of these cold phenomena [2, 3]. Cold waves, characterized by prolonged periods of decreasing temperatures, and extreme cold events, marked by sudden drops and brief periods of intense cold, present significant risks to health [4]. In South Korea, although average winter temperatures have remained relatively stable over the past decade, there has been an increase in the number of days with temperatures of −5 °C and −10 °C, suggesting a trend toward more extreme cold events [5]. Consequently, cold-related illnesses and the relative risk of mortality due to cold waves have risen annually [6].

The contemporary population is generally well-protected in winter, wearing clothing with sufficient insulation and benefitting from reliable indoor heating systems in urbanized areas. Nevertheless, as mentioned earlier, the increasing frequency of unexpected cold waves likely contributes to rising rates of winter illnesses, including colds, and the prevalence of cold-related health conditions. In particular, sudden gusts of wind, which are common in urban areas with dense high-rise buildings, often cause discomfort for pedestrians [7]. Analitis et al. found that a 1 °C decrease in temperature correlates with a 1.35% increase in daily mortality rates, underscoring the severe impact of cold waves [8]. Many previous studies have demonstrated that the addition of wind exacerbates cold stress [9, 10]. However, urban people who live in temperate climates are accustomed to mild cold with varying wind speeds, and relatively few studies have examined the effects of wind speed in mild cold on individuals wearing winter clothing, especially compared to studies conducted in severe cold environments.

The wind chill index (WCI) or wind chill temperature (WCT, *t*_wc_), which combines air temperature and wind speed to measure cold stress, provides a standardized way to assess cold exposure [11]. It categorizes cold stress into four levels: Uncomfortably cold (−10 to −24 °C), very cold (risk of skin freezing) (−25 to −34 °C), bitterly cold (freezing in 10 min) (−35 to − 9 °C), and extremely cold (freezing in 2 min) (below −60 °C) [12]. Typically, the wind speeds experienced in urban areas of temperate regions such as Korea and Japan reach a maximum of around 7 m·s−^1^. Consequently, winters in these regions fall within the “uncomfortably cold” category on the WCT index [13–15]. The ISO 11079 [16] standard outlines a methodology for assessing cold stress in individuals exposed to cold environments using the WCT. Health and safety organizations in countries like the USA, Canada, and Northern Europe also utilize the WCI to provide guidelines on appropriate clothing insulation to withstand winter conditions comfortably. Despite these standards, there is a lack of quantified data on the specific cold stress experienced by residents of temperate regions with mild winter climates, such as South Korea or Japan. In particular, there is limited research on the psychophysiological responses of individuals to wind speeds in mild cold environments. Therefore, this study examined both the physiological and subjective responses to wind speeds up to 7 m·s^−1^ in mild cold, considering anthropometric characteristics of individuals.

## Methods

### Subjects

Ten males with no history of cold-related illnesses participated in the experiment (mean ± SD: 23.9 ± 3.3 years in age, 175.8 ± 4.9 cm in height, 74.4 ± 7.0 kg in body weight, 24.0 ± 1.8 kg·m^−2^ in body mass index [BMI], 1.94 ± 0.11 m^2^ in body surface area, and 19.8 ± 3.4% in total body fat; 4 overweight and 6 normal BMI, Table 1). According to the World Health Organization (WHO) guidelines [17], subjects with a BMI of 25 or higher were classified as overweight in the Asia–Pacific region. All subjects were nonsmokers and were not professional or competitive athletes. The experimental protocol was approved by the Institutional Review Board at Seoul National University (IRB No. 2108/002–017).

**Table 1:** Anthropometric characteristics of the subjects

Subject no.	Age (y)	Height (cm)	Body weight (kg)	Body mass index, BMI (kg·m^−2^)	Body fat mass (kg)	Body surface area, BSA (m^2^)	Total body fat (%)
1	22	178.0	68.5	21.6	10.4	1.89	15.2
2	22	169.0	72.2	**25.3**	19.0	1.86	26.3
3	28	184.0	86.2	**25.5**	17.0	2.14	19.7
4	21	175.0	70.5	23.0	12.5	1.89	17.8
5	22	168.0	63.0	22.3	10.3	1.75	16.3
6	26	176.4	80.8	**26.0**	16.5	2.02	20.4
7	22	173.0	81.3	**27.2**	17.8	1.99	21.9
8	22	178.0	71.5	22.6	12.0	1.93	16.8
9	23	176.0	72.2	23.3	15.2	1.92	21.0
10	31	181.0	77.9	23.8	17.7	2.02	22.8
Mean	23.9	175.8	74.4	24.0	14.8	1.94	19.8
SD	3.3	4.9	7.0	1.8	3.3	0.11	3.4

Body surface area was calculated using Lee and Choi’s formula [18]

### Experimental conditions and clothing

**Fig. 1:**
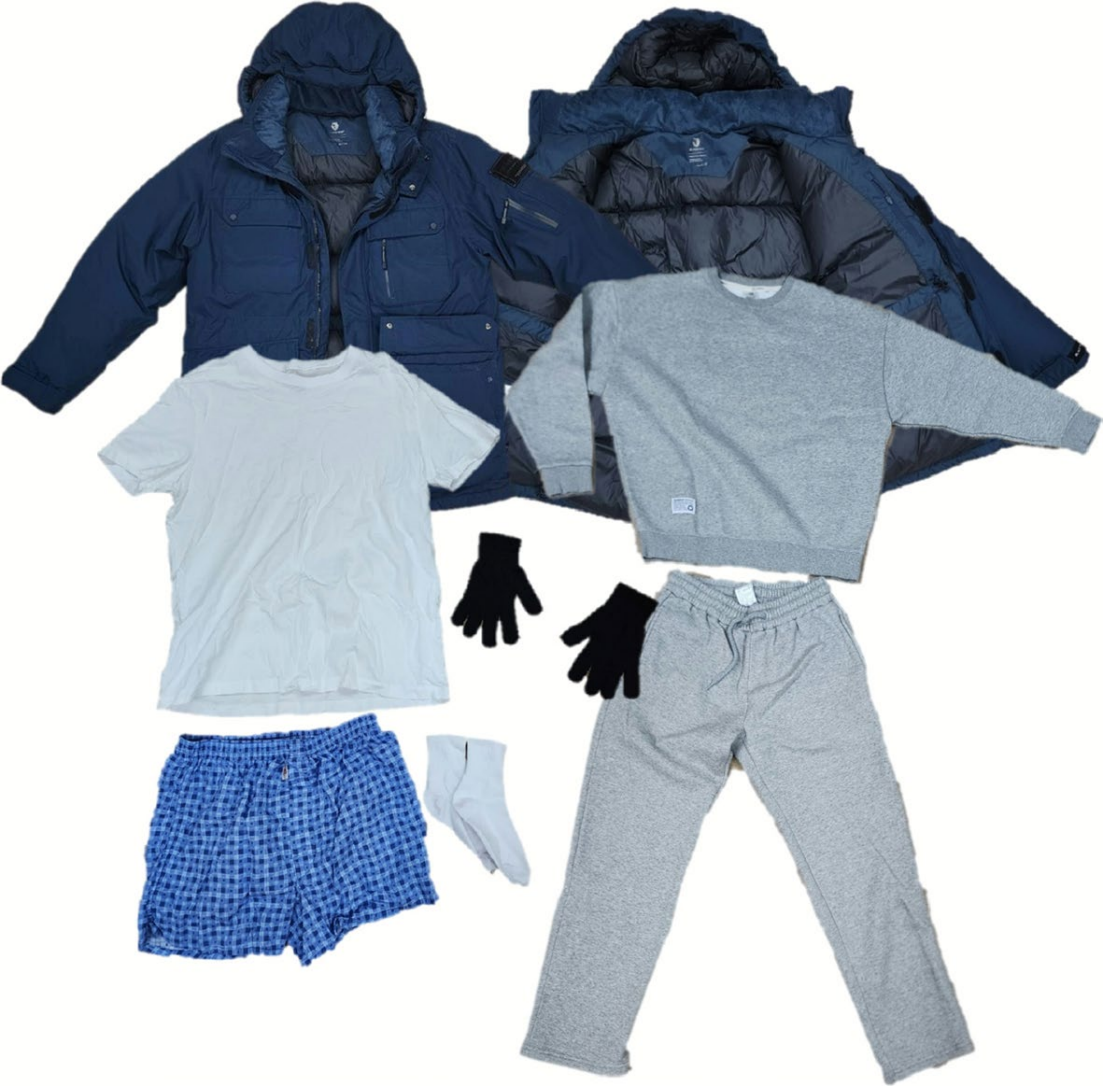
Experimental clothing

Subjects underwent all the following four wind speed conditions: 0, 2, 4.5, and 7 m·s^−1^. In typical urban environments, daily wind speeds are generally below 7 m·s^−1^ [13]. In the present study, the artificial wind was generated using two large fans, each equipped with 75-cm diameter blades and positioned at heights of 165 cm and 90 cm, respectively. The wind intensity was controlled by varying the revolutions per minute (RPM) of the fans. Wind speed measurements were taken at three different height levels (50 cm, 100 cm, and 150 cm above the floor) using an anemometer (KA-23, Kanomax, Japan). Each measurement was repeated three times, and the average of these readings was used for each wind speed condition. All trials were conducted in a climate chamber at an air temperature of −5 °C. The experimental clothing consisted of a brief, long pants, a short-sleeve T-shirt, a long-sleeve T-shirt, a padded jacket (80% duck down and 20% feathers), single-layer polyester-acrylic blend finger gloves (34 g), socks, and running shoes (3234 g in total clothing mass, excluding the shoes, Fig. 1). The padded jacket included an attached hood, which was worn during exposure. The hood covered the back of the neck and head up to the hairline. The experimental clothing ensemble corresponded to typical winter clothing in Korea. The padded jacket was available in three sizes: M, L, and XL, allowing for selection according to the subjects’ body sizes. The thermal insulation of the experimental clothing ensemble was measured using a thermal manikin (Newton, 20 zones, Thermetrics Inc., USA) in a standard environment (21 °C with 50%RH, wind speed < 0.2 m·s^−1^) and the four experimental conditions. For the standard condition, the thermal insulation of the air layer (*I*_a_) was 0.62 clo, and the total thermal insulation (*I*_T_) was 2.08 clo. The total clothing insulation for the four wind speed conditions (0, 2, 4.5, 7 m·s^−1^) at the air temperature of −5 °C was 2.04, 1.42, 1.12, and 0.80 clo, respectively (100%, 70%, 55%, and 39%). The wind chill indices (WCI) and wind chill temperatures (WCT) corresponding to each condition were calculated to be 397, 859, 1087, and 1175 W·m^−2^, and −5 °C, −8 °C, −10 °C, and −12 °C, respectively. Minimal required clothing insulation (*IREQ*_min_) was 2.7, 2.7, 2.8, and 2.8 clo for the WCT of −5 °C, −8 °C, −10 °C, and −12 °C, respectively [16].

### Experimental protocol and measurements

A trial consisted of a 10-min rest followed by a 60-min walking on a treadmill at 4 km·h^−1^ and a 10-min recovery. Wind was activated during the 60-min walking phase only. All experiments were conducted during the same hours (9:00 to 11:00 AM) to minimize the effects of circadian rhythms. The experiment was terminated if rectal temperature fell below 36.2 °C or exceeded 39.2 °C, if their heart rate reached 90% of the maximum heart rate, or if the subject chose to withdraw.

Rectal temperature (*T*_re_) was recorded using a portable rectal thermometer (LT8A, Gram Corps., Japan) with a thermistor sensor, at a depth of 15–16 cm within the rectum. Auditory canal temperature (*T*_ac_) was measured with the same device and a sensor at a depth of 2–3 cm within the ear canal. Gastrointestinal temperature (*T*_pill_) was measured using a telemetric pill and a data logger (e-Celsius, France). To account for the transit time of the pill through the esophagus, subjects ingested the pill 4 h prior to every trial. Skin temperature (*T*_sk_) was measured using the identical thermometer (LT8A, Gram Corps., Japan) at the following 11 skin sites: the forehead (*T*_forehead_), chest (*T*_chest_), upper back (*T*_back_), abdomen (*T*_abdomen_), forearm (*T*_forearm_), dorsal hand (*T*_hand_), thigh (*T*_thigh_), calf (*T*_calf_), dorsal foot (*T*_foot_), the 3rd finger (*T*_finger_), and big toe (hallux) (*T*_toe_). All the temperature measurements were recorded every 5 s for 80 min. Mean skin temperature was calculated using the formula from Hardy and DuBois (1938): Mean *T*_sk_ = 0.07*T*_forehead_ + 0.35(*T*_chest_ + *T*_back_ + *T*_abdomen_)/3 + 0.14*T*_forearm_ + 0.05*T*_hand_ + 0.19*T*_thigh_ + 0.13*T*_calf_ + 0.07*T*_foot_. Cold Strain Index (CSI) was calculated using rectal temperature (*T*_re_) and mean skin temperature ($\overline{T}$_sk_): CSI = 6.67(*T*_re* t*_−*T*_re 0_) · (35 − *T*_re 0_)^−1^ + 3.33 ($\overline{T}$_sk *t*_−$\overline{T}$_sk 0_) · (20−$\overline{T}$_sk 0_)^−1^. If *T*_re *t*_ is greater than *T*_re 0_, then the term (*T*_re *t*_ − *T*_re 0_) is set to 0.

Clothing microclimate temperature and humidity were monitored using portable recorders (Thermo Recorder TR-72U, T&D, Japan) in the innermost air layer over the chest and back areas. Body weight was recorded three times each, immediately before and after the experiment, using a scale (ID2, Sartorius, Mettler-Toledo, Germany; unit: grams). Total body mass loss was assessed with the difference. Energy expenditure was continuously monitored for 80 min using a metabolic analyzer (Quark CPET, COSMED, Italy) based on the breath-by-breath method. Heart rate (HR) was recorded every 1 s using a portable heart rate monitor (RS400/800, Polar, Finland). Blood pressure (BP) was measured using a portable sphygmomanometer, three times each, at rest, immediately following exercise, and the recovery period.

Thermal sensation was assessed using a 9-point categorical scale with intervals of 0.5 points [4 very hot, 3 hot, 2 warm (slightly hot), 1 slightly warm, 0 not both, −1 slightly cool, −2 cool, −3 cold, −4 very cold]. Thermal comfort was evaluated using a 7-point categorical scale with intervals of 0.5 points [3 very comfortable, 2 comfortable, 1 a little comfortable, 0 neither, −1 a little uncomfortable, −2 uncomfortable, −3 very uncomfortable]. Sensation to wind was evaluated using a 6-point categorical scale with intervals of 0.5 points [5 very, very strong, 4 very strong, 3 strong, 2 slightly strong, 1 weak, 0 almost none]. Thermal sensation and thermal comfort were recorded separately for the whole body, hands, and feet. For shivering sensation, subjects indicated the specific body regions where they felt shivered, based on a predetermined body segmentation chart (21 regions + fingers [#22] + toes [#23]) [18]). All subjective responses were recorded every 10 min.

### Data analysis

Statistical analyses were conducted using IBM SPSS Statistics version 26.0. Data were presented as mean ± standard deviation (SD) or standard error (SE). Normality was assessed using the Shapiro–Wilk test. Differences among the four wind speed conditions were analyzed using repeated measures ANOVA, followed by Bonferroni correction for multiple comparisons. For nonparametric variables, the Friedman test was used to evaluate differences across the four conditions. The *P*-values of less than 0.05 were considered statistically significant.

## Results

### Rectal, gastrointestinal, and auditory canal temperatures

**Fig. 2:**
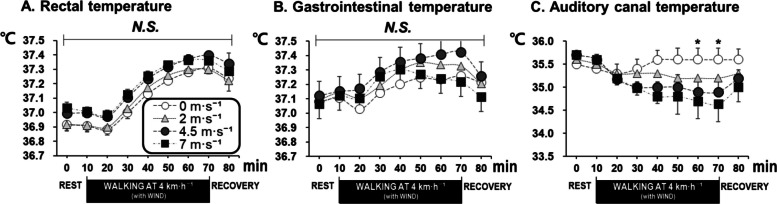
Time courses of rectal temperature (**A**), gastrointestinal temperature (**B**), and auditory canal temperature (**C**) for the four wind speed conditions (mean ± SE). Note: a and b represent significantly classified groups by pairwise comparisons with the Bonferroni correction. *N.S.* means no significance, *P < 0.05

No significant differences in *T*_re_ among the four wind speed conditions during rest, exercise, and recovery were found (Fig. 2A). *T*_pill_ showed no differences among the four wind conditions (Fig. 2B). *T*_ac_ gradually increased for the 0 m/s wind condition during the 60-min walking, while for the three wind conditions, *T*_ac_ gradually decreased during the walking. There was a significant difference between the wind speed conditions of 0 m⋅s^−1^ and − 7 m⋅s^−1^ (Fig. 2C, *P* < 0.05).

### Skin temperature

*_T*_sk_ at rest showed no significant differences among the four wind conditions (Table 2). During walking, *_T*_sk_ gradually decreased, reaching 26.2 to 29.6 °C at the end of the exposure, and showed significant group differences (*P* < 0.001, Table 2). There were no significant differences in chest, back, forearm, and calf temperatures among the four wind conditions, while forehead, abdomen, thigh, hand, finger, foot, and toe temperatures showed group differences among the four wind conditions, especially at the end of walking (all Ps < 0.05, Table 2). In particular, hand and finger temperatures gradually decreased during exercise for all four wind conditions, showing 13.7 ± 0.3 °C and 12.9 ± 0.8 °C, respectively, at the end of exercise for the 7 m·s^−1^ wind condition. Thigh, foot, and toe temperatures also decreased during exercise with significant differences among the four conditions (Table 2).

**Table 2:** Mean and regional skin temperatures for the four wind speed conditions

Skin temperature	Wind speed (m·s^−1^)	Rest (no wind)		Exercise (wind)		Recovery (no wind)	
		Initial 3 min	Last 3 min	Initial 3 min	Last 3 min	Initial 3 min	Last 3 min
Mean skin temp.	0	31.8 ± 0.1	31.2 ± 0.1	30.7 ± 0.2	29.6 ± 0.3d	29.8 ± 0.3d	29.9 ± 0.3c
	2	31.4 ± 0.3	30.8 ± 0.3	30.3 ± 0.3	28.0 ± 0.4c	28.4 ± 0.4c	29.1 ± 0.3b
	4.5	31.7 ± 0.3	31.1 ± 0.3	30.5 ± 0.3	27.2 ± 0.3b	27.6 ± 0.3b	28.6 ± 0.3b
	7	31.5 ± 0.4	31.1 ± 0.2	30.1 ± 0.3	26.2 ± 0.4a	26.9 ± 0.3a	28.0 ± 0.3a
*P*-value		0.699	0.402	0.315	< 0.001	< 0.001	< 0.001
Forehead	0	33.1 ± 0.4	33.3 ± 0.3	33.0 ± 0.3	32.4 ± 0.4b	32.5 ± 0.4b	33.1 ± 0.3b
	2	32.8 ± 0.2	32.7 ± 0.3	31.8 ± 0.5	29.0 ± 1.0a	29.8 ± 0.8a	31.8 ± 0.5a
	4.5	33.0 ± 0.4	33.0 ± 0.5	32.0 ± 0.7	29.4 ± 1.0a	30.2 ± 0.9a	32.6 ± 0.4ab
	7	33.0 ± 0.3	33.4 ± 0.2	31.8 ± 0.4	27.2 ± 1.2a	28.9 ± 0.9a	32.2 ± 0.4a
*P*-value		0.849	0.345	0.195	< 0.001	0.001	0.025
Upper back	0	34.2 ± 0.2	34.1 ± 0.2	34.0 ± 0.1	34.1 ± 0.2	34.1 ± 0.2	34.1 ± 0.3
	2	34.2 ± 0.2	34.1 ± 0.2	34.1 ± 0.2	33.9 ± 0.2	34.0 ± 0.3	34.1 ± 0.3
	4.5	34.0 ± 0.2	34.0 ± 0.3	34.0 ± 0.2	34.1 ± 0.3	34.1 ± 0.3	34.1 ± 0.3
	7	34.0 ± 0.3	34.1 ± 0.3	33.9 ± 0.2	34.1 ± 0.6	34.1 ± 0.3	34.1 ± 0.3
*P*-value		0.682	0.881	0.876	0.557	0.503	0.066
Abdomen	0	33.1 ± 0.2	33.3 ± 0.2	33.2 ± 0.2	33.2 ± 0.3d	33.2 ± 0.3d	33.6 ± 0.3d
	2	32.9 ± 0.4	33.1 ± 0.4	33.0 ± 0.3	31.4 ± 0.5c	31.5 ± 0.5c	32.1 ± 0.5c
	4.5	32.9 ± 0.4	33.1 ± 0.4	32.9 ± 0.4	29.4 ± 0.6b	29.5 ± 0.7b	30.6 ± 0.7b
	7	32.9 ± 0.3	33.1 ± 0.4	32.6 ± 0.4	26.7 ± 0.9a	26.9 ± 0.9a	28.2 ± 0.9a
*P*-value		0.929	0.934	0.507	< 0.001	< 0.001	< 0.001
Hand	0	30.1 ± 0.4	28.6 ± 0.3	27.9 ± 0.3	23.5 ± 1.2d	23.5 ± 1.2d	24.4 ± 1.2d
	2	29.4 ± 0.9	28.2 ± 0.9	27.4 ± 0.9	19.4 ± 1.5c	19.9 ± 1.6c	21.5 ± 1.8c
	4.5	30.3 ± 0.3	29.3 ± 0.5	28.1 ± 0.5	16.3 ± 1.1b	16.5 ± 1.1b	18.5 ± 1.0b
	7	30.4 ± 0.3	29.0 ± 0.5	27.5 ± 0.5	**13.7 ± 0.3a**	**14.2 ± 0.3a**	16.0 ± 0.7a
*P*-value		0.452	0.398	0.708	< 0.001	< 0.001	< 0.001
Third finger	0	30.1 ± 0.8	28.4 ± 1.3	24.9 ± 1.2	23.7 ± 2.4c	26.0 ± 2.1c	28.3 ± 1.8b
	2	27.0 ± 2.2	25.8 ± 2.5	23.2 ± 2.3	19.5 ± 2.2b	21.3 ± 2.4b	24.7 ± 2.1b
	4.5	28.4 ± 1.6	26.4 ± 2.2	23.0 ± 2.1	15.5 ± 2.1a	17.2 ± 2.1ab	23.9 ± 2.0b
	7	28.8 ± 1.1	26.4 ± 1.8	22.6 ± 1.7	**12.9 ± 0.8a**	**14.5 ± 0.7a**	20.4 ± 2.0a
*P*-value		0.301	0.545	0.614	< 0.001	< 0.001	0.003
Thigh	0	30.2 ± 0.6	28.9 ± 0.8	27.7 ± 0.5b	25.0 ± 0.6c	25.8 ± 0.5d	26.6 ± 0.6c
	2	29.0 ± 0.7	28.1 ± 0.7	26.8 ± 0.6a	21.7 ± 0.8b	22.5 ± 0.8c	24.4 ± 0.8b
	4.5	29.2 ± 0.4	27.9 ± 0.5	26.4 ± 0.5a	18.3 ± 0.6a	19.7 ± 0.7b	22.4 ± 0.8a
	7	28.1 ± 1.3	27.6 ± 0.4	24.6 ± 1.3a	16.3 ± 1.8a	18.4 ± 1.4a	21.4 ± 1.2a
*P*-value		0.267	0.140	0.030	< 0.001	< 0.001	< 0.001
Foot	0	31.8 ± 0.4	30.7 ± 0.4	30.4 ± 0.4	29.6 ± 1.1b	29.6 ± 1.1c	29.3 ± 1.0c
	2	30.6 ± 0.7	29.6 ± 0.8	29.3 ± 0.8	27.6 ± 1.5a	27.7 ± 1.4b	27.5 ± 1.4b
	4.5	31.7 ± 0.4	30.8 ± 0.5	30.4 ± 0.5	26.0 ± 1.3a	26.1 ± 1.3ab	26.0 ± 1.2ab
	7	31.2 ± 0.9	31.3 ± 0.3	30.8 ± 0.3	26.2 ± 1.4a	26.3 ± 1.4a	26.0 ± 1.3a
*P*-value		0.563	0.156	0.217	< 0.001	< 0.001	< 0.001
Toe	0	28.2 ± 1.2	25.8 ± 1.2	24.9 ± 1.2	22.6 ± 2.1c	22.6 ± 2.0c	22.1 ± 2.0b
	2	26.2 ± 1.4	24.4 ± 1.5	23.5 ± 1.5	19.8 ± 2.3b	19.8 ± 2.3b	19.1 ± 2.2a
	4.5	28.2 ± 1.1	26.5 ± 1.3	25.5 ± 1.3	17.4 ± 2.0a	17.3 ± 1.9a	16.9 ± 2.0a
	7	27.2 ± 1.6	26.5 ± 0.7	25.6 ± 0.7	17.6 ± 1.8ab	17.6 ± 1.9ab	17.0 ± 1.7a
*P*-value		0.677	0.358	0.356	< 0.001	< 0.001	0.001

Values are presented as mean ± SE of 10 subjects (°C). a, b, c, and d represent significantly classified groups by pairwise comparisons with the Bonferroni correction

### Heart rate, energy expenditure, blood pressure, and total body mass loss

For heart rate, there were no significant differences among the four wind speed conditions. The average heart rate ranged from 71 to 74 bpm at rest, from 95 to 101 bpm at the end of the 60-min walking, and returned to resting levels with an average of 73 to 77 bpm during recovery. Energy expenditure was calculated as 261 to 290 kcal h^−1^ on average, with no significant difference among the four wind conditions. No significant differences were observed in both diastolic and systolic blood pressure among the four wind speed conditions, and blood pressure remained within the normal range throughout (Table 3). Total body mass loss (insensible perspiration) during the 80-min exposure averaged between 186 and 200 g/trial, with no significant differences among the four wind speed conditions.

**Table 3:** Systolic and diastolic blood pressures under the four wind speed conditions

Phase	0 m·s^−1^		2 m·s^−1^		4.5 m·s^−1^		7 m·s^−1^	
	Systolic	Diastolic	Systolic	Diastolic	Systolic	Diastolic	Systolic	Diastolic
Pre-exposure (baseline)	118 ± 7	82 ± 9	119 ± 7	82 ± 9	122 ± 11	80 ± 9	121 ± 9	81 ± 6
End of rest	122 ± 8	88 ± 9	121 ± 9	85 ± 8	123 ± 8	85 ± 11	126 ± 8	87 ± 8
Immediately post-exercise	122 ± 8	83 ± 8	123 ± 10	84 ± 12	125 ± 11	83 ± 9	127 ± 8	87 ± 7
End of recovery	120 ± 6	83 ± 8	122 ± 7	84 ± 11	122 ± 8	84 ± 10	125 ± 8	89 ± 9

Values are presented as mean ± SD of 10 subjects (mmHg). Repeated-measures ANOVA revealed no significant main effect of wind speed on either systolic or diastolic blood pressure across all phases

### Clothing microclimate

**Fig. 3:**
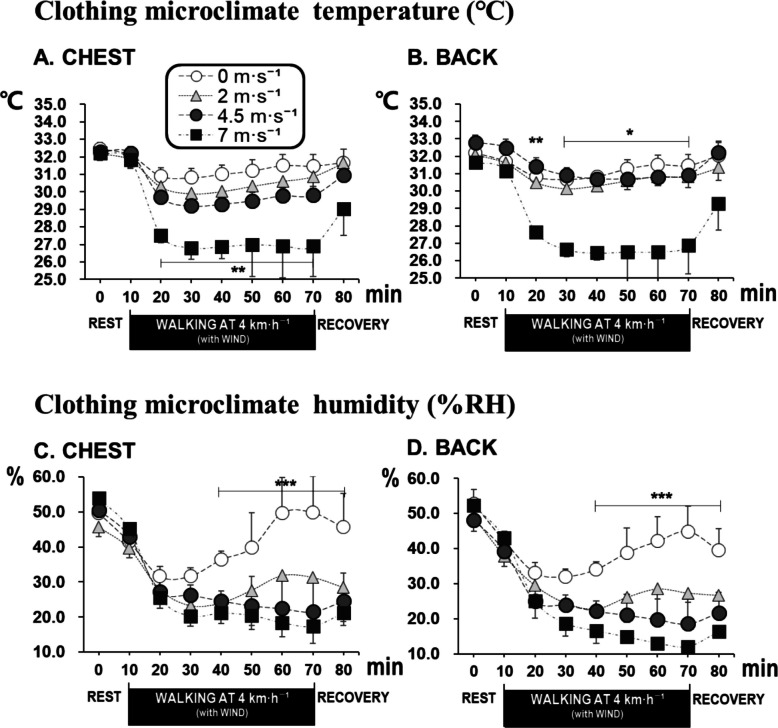
Time courses of chest (**A**) and back (**B**) clothing microclimate temperatures and chest (**C**) and back (**D**) clothing microclimate humidity for the four wind speed conditions (mean ± SE). Note: a, b, and c represent significantly classified. *P < 0.05, **P < 0.01, and ***P < 0.001

Significant differences in clothing microclimate temperature on the chest among the four wind conditions were found for the 60-min walking, with temperatures decreasing to an average of 26.9 °C for the 7 m·s^−1^ condition (Fig. 3A). These differences persisted until the early recovery period (Fig. 2A). Clothing microclimate temperature on the upper back showed similar results as those from the chest (Fig. 3B). During the 60-min walking, the four wind conditions were divided into statistically distinct groups: no wind = 2 m·s^−1^ wind > 7 m·s^−1^ wind. The 4.5 m·s^−1^ wind condition was statistically classified as the same as either the 2 ms^−1^ or the 7 ms^−1^ wind conditions (Fig. 3). For clothing microclimate humidity on the chest, significant differences were observed among the four wind conditions, during the latter part of the 60-min walking, with humidity decreasing to an average of 17%RH at a wind speed of 7 m·s⁻^1^ (Fig. 3C). Clothing microclimate humidity on the upper back showed similar results as those from the chest (Fig. 3D). During the latter part of the 60-min walking, the four wind conditions were divided into three statistically distinct conditions: no wind > 2 m·s^−1^ wind > 7 m·s^−1^ wind. The 4.5 m·s^−1^ wind group was statistically classified as the same as either the 2 m·s^−1^ or the 7 m·s^−1^ wind conditions.

### Subjective responses

**Fig. 4:**
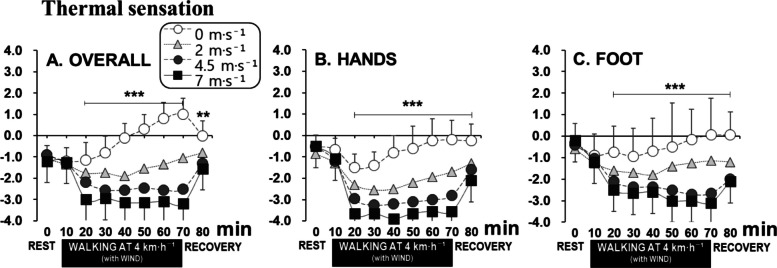
Time courses of overall (**A**), hand (**B**), and foot (**C**) thermal sensation for the four wind speed conditions (mean ± SD). Note: a, b, and c represent significantly classified groups. **P < 0.01, ***P < 0.001

Significant differences in overall thermal sensation were observed among the four wind conditions after just 10 min of wind exposure. Additionally, the 4.5 m·s^−1^ condition exhibited significant differences in thermal sensation compared to both the 0 m·s^−1^ and 7 m·s^−1^ conditions. After 60 min of exposure to −5 °C with 7 m·s^−1^ wind, the overall body sensation averaged −3.2, corresponding to a “cold” rating (Fig. 4A). Hand and foot thermal sensations showed similar tendencies as those from overall thermal sensations (Figs. 4B, 3C). At the 40-min exposure of the 7 m·s^−1^ wind condition, the hand thermal sensation was −3.9 on average, categorized as “very cold,” which was approximately 1 point lower than that recorded for the feet or overall body (Figs. 4B, 3C).

**Fig. 5:**
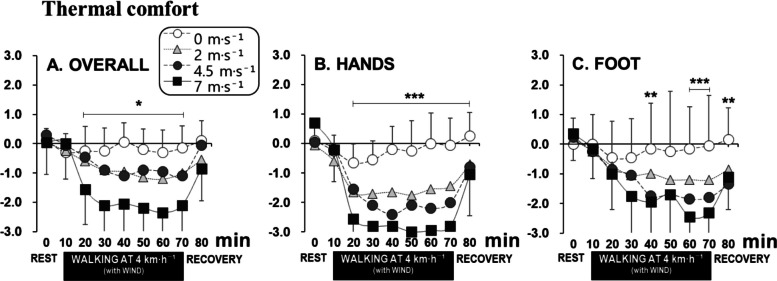
Time courses of overall (**A**), hand (**B**), and foot (**C**) thermal comfort for the four wind speed conditions (mean ± SD). Note: *P < 0.05, **P < 0.01, ***P < 0.001

**Fig. 6:**
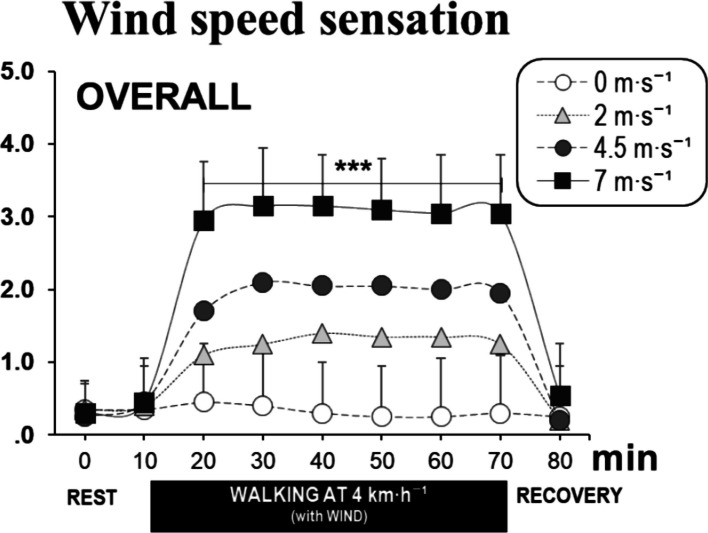
Time courses of overall wind speed sensation for the four wind speed conditions (mean ± SD). Note: *P < 0.05, **P < 0.01, ***P < 0.001

Significant differences in overall thermal comfort were observed between the 0 m·s^−1^ and 7 m·s^−1^ wind conditions after 10 min of wind exposure. Thermal comfort under the 4.5 m·s^−1^ or 2 m·s^−1^ wind conditions showed significant differences compared to that at 0 m·s^−1^ or 7 m·s^−1^ conditions (Fig. 5A). After 70 min of exposure to −5 °C for the 7 m·s^−1^ wind condition, the overall thermal comfort averaged −2.1, indicating a rating of “uncomfortable.” Similarly, hand and foot thermal comfort followed the overall body trend, though discomfort was more pronounced in the hands. By the 50-min exposure for the 7 m·s^−1^ wind condition, the average hand comfort rating was −3.0, categorized as “very uncomfortable,” which was approximately 1 point lower than the comfort ratings for the feet (average −1.7) or the entire body (average −2.2) (Fig. 5). Significant differences were observed between the 0 m·s^−1^, 2 m·s^−1^, 4.5 m·s^−1^, and 7 m·s^−1^ conditions. After 60-min of exposure, the wind sensation was rated as an average of 0.3, 1.3, 2.0, and 3.1 points, respectively, corresponding to “none,” “weak,” “moderately strong,” and “strong” sensations (Fig. 6).

The total number of shivering sensations reported was 20, 40, 55, and 114 times for the 0 m·s^−1^, 2 m·s^−1^, 4.5 m·s^−1^, and 7 m·s^−1^ conditions, respectively. The most frequently reported body region with shivering sensation was the hands (without fingers) for all the four wind conditions, followed by the palms, feet, fingers, and thighs. For the 7 m·s^−1^ condition, shivering was predominantly reported in the hands, palms, thighs, and feet. Additionally, for the 7 m·s^−1^ condition, shivering was reported in regions where it was not observed under the other conditions, such as the forehead, chest, calves, and buttocks. We plotted the shivering frequency from the four conditions by two subject groups divided into the overweight (four individuals) and normal group (six individuals). The frequency of reported shivering sensations was smaller for the overweight group (13, 6, 14, and 51 times for the 0 m·s^−1^, 2 m·s^−1^, 4.5 m·s^−1^, and 7 m·s^−1^ conditions, respectively) compared to the normal group (7, 34, 41, and 63 times) for all the three wind conditions. When analysis was restricted to the wind conditions, the total number of shivering events did not differ significantly between the normal BMI and overweight groups (*P* = 0.319). Similarly, BMI was not correlated with total shivering frequency (*ρ* = 0.115, *P* = 0.546). In contrast, body surface area (BSA) showed a significant positive correlation with total shivering frequency (*ρ* = 0.534, *P* = 0.002).

### Relationships between Cold Strain Index (CSI), subjective responses, hand temperature, and physical properties

**Fig. 7:**
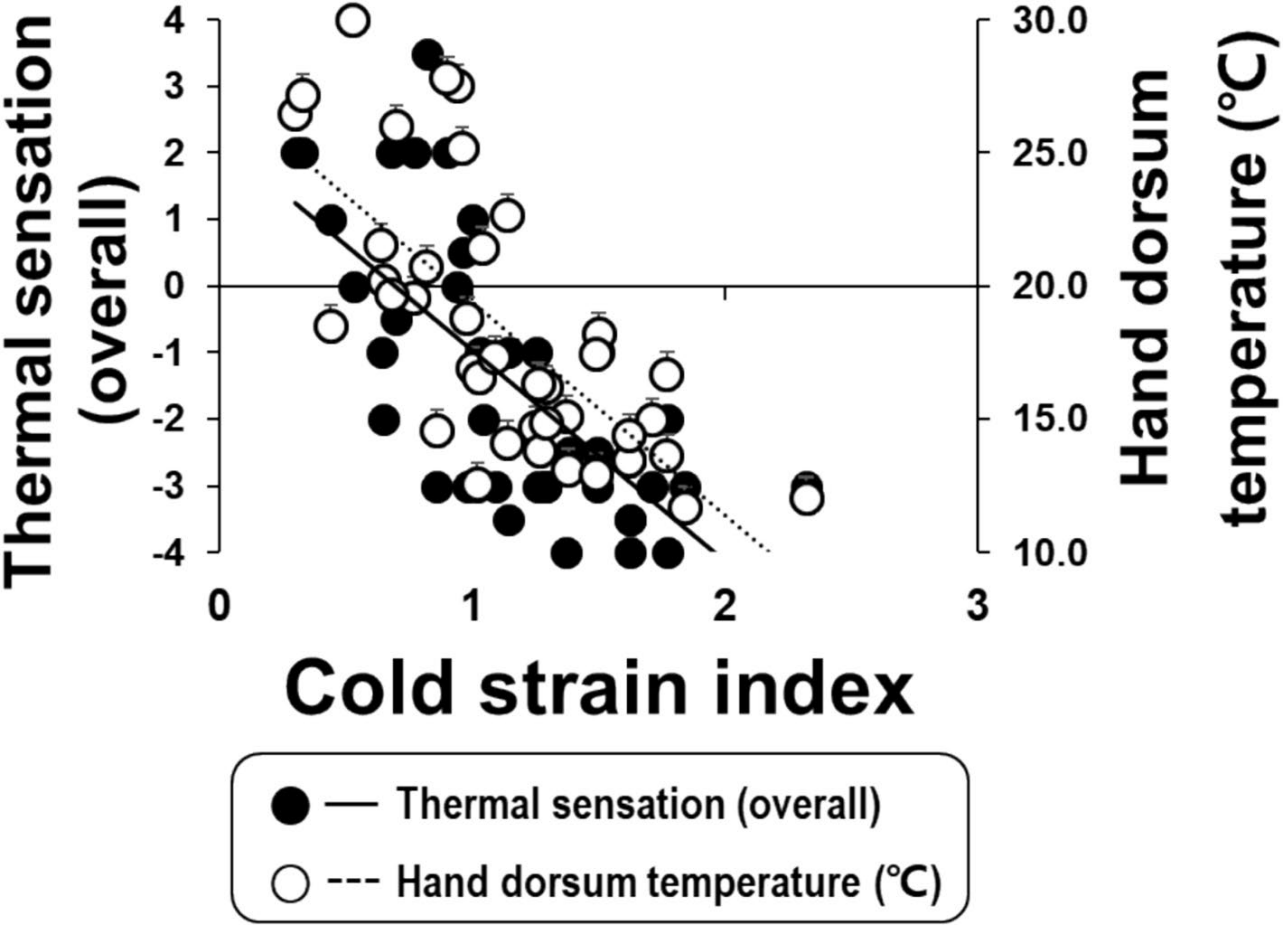
Relationships between Cold Strain Index (CSI) and overall thermal, hand dorsum temperature at the end of exercise for the four air temperature conditions

Cold Strain Index (CSI) averaged between 0.67 and 1.54, with significant differences among the four wind conditions (Table 4). There were negative correlations between CSI and overall thermal sensation or hand temperature (rho = −0.718 for thermal sensation, rho = −0.747 for hand temperature, all *Ps* < 0.001; Fig. 7). Hand temperature was highly correlated with overall thermal comfort, as well as hand thermal sensation and comfort. Body surface area (BSA) showed a significant negative correlation with overall thermal sensation and comfort, as well as hand thermal sensation and comfort at the end of the exercise. Additionally, toe temperature exhibited a significant negative correlation with BSA (*ρ* = −0.585, *P* = 0.001, Table 5). Hand temperature was highly correlated with overall thermal sensation and comfort, hand thermal sensation and comfort, and overall wind speed sensation (Table 5). BMI and BSA per unit body weight showed a significant negative and positive correlation, respectively, only with overall thermal comfort, while total body fat (%) did not show any significant relationships with psychological responses.

**Table 4:** Cold Strain Index (CSI) for the four wind speed conditions

Wind speed (m·s^−1^)	Rest (no wind)		Exercise (wind)		Recovery (no wind)	
	Initial 3 min	Last 3 min	Initial 3 min	Last 3 min	Initial 3 min	Last 3 min
0	0.07 ± 0.06	0.28 ± 0.06	0.43 ± 0.06	0.67 ± 0.06a	0.62 ± 0.06a	0.58 ± 0.06a
2	0.06 ± 0.08	0.30 ± 0.08	0.46 ± 0.08	1.03 ± 0.08b	0.92 ± 0.08ab	0.73 ± 0.08ab
4.5	0.07 ± 0.06	0.25 ± 0.06	0.48 ± 0.06	1.34 ± 0.06bc	1.21 ± 0.06c	0.94 ± 0.06b
7	0.07 ± 0.08	0.22 ± 0.08	0.54 ± 0.08	1.54 ± 0.08c	1.33 ± 0.08bc	1.04 ± 0.08b
*P*-value	0.797	0.614	0.493	< 0.001	< 0.001	0.001

Note 1: Values are presented as mean ± SE. Note 2: a, b, and c represent significantly classified groups by pairwise comparisons with the Bonferroni correction

**Table 5:** Relationships between morphological and physiological properties and subjective responses

		Thermal sensation			Thermal comfort			Wind sensation	Body surface area, BSA* (m^2^)
		Overall	Hands	Feet	Overall	Hands	Feet	Overall	
BMI (kg·m^−2^)	*ρ*	−0.005	−0.005	−0.030	−0.335	−0.151	−0.216	−0.015	0.628
	*P*	0.974	0.974	0.854	**0.035**	0.353	0.182	0.925	** < 0.001**
BSA* (m^2^)	*ρ*	−0.334	−0.360	−0.341	−0.341	−0.312	−0.447	0.133	1.000
	*P*	**0.035**	**0.023**	**0.032**	**0.032**	**0.050**	**0.004**	0.415	-
BSA·body weight^−1^ (cm^2^·kg^−1^)	*ρ*	0.063	0.076	0.114	0.352	0.193	0.297	−0.031	−0.677
	*P*	0.699	0.639	0.483	**0.026**	0.232	0.063	0.851	**< 0.001**
Hand temp. (°C)	*ρ*	0.728	0.681	0.509	0.485	0.673	0.492	−0.696	−0.230
	*P*	**< 0.001**	**< 0.001**	**< 0.001**	**0.002**	**< 0.001**	**0.001**	**< 0.001**	0.221
Toe temp. (°C)	*ρ*	0.564	0.676	0.658	0.297	0.570	0.686	−0.389	−0.585
	*P*	**0.001**	**< 0.001**	**< 0.001**	0.112	**< 0.001**	**< 0.001**	**0.034**	**0.001**

*ρ* Spearman correlation coefficient, *P* P-value

*Body surface area was calculated using Lee and Choi’s formula [18]

## Discussion

### Is the typical winter clothing sufficient to protect the body from strong winds in mild winter temperatures?

This study found that typical winter clothing (*I*_T_ = 2.1 clo) effectively maintained core body temperature in mild winter temperature (−5 °C) with the strong wind of 7 m·s^−1^ (Fig. 2 A, B). Blood pressure also remained stable across all wind conditions (Table 3), indicating no significant circulatory stress under the mild cold exposure. In fact, during the 60-min walk, *T*_re_ increased by an average of 0.4 to 0.5 °C, and *T*_pill_ increased by an average of 0.2 °C. This indicates that the heat generated from physical activity exceeded the heat lost in the cold and windy conditions. Meanwhile, *T*_ac_ showed significant differences among wind conditions, decreasing progressively as wind speed increased. As is well known, *T*_ac_ is strongly affected by ambient air and wind speed, suggesting that *T*_ac_ may not be a reliable substitute for core body temperature in windy and cold conditions [19].

Although the upper back temperature showed little variation among wind conditions (Table 2), the clothing microclimate temperature at the back decreased significantly at 7 m·s^−1^ (Fig. 3). This indicates that while the thick insulation and limited air exchange protected the upper back region from convective cooling, internal air circulation within the multilayered clothing still promoted some heat loss from the clothing microenvironment. A movement-induced bellows effect may have further reduced the clothing microenvironment by enhancing convective heat loss [20]. These region-specific responses are consistent with previous findings that the posterior trunk tends to preserve thermal stability due to both anatomical blood flow patterns and clothing coverage effects [20, 21].

However, the mean skin temperature dropped below 27 °C, and the finger temperature dropped below 13 °C after 60-min walking even while wearing gloves (Table 2). Finger temperatures below approximately 13 °C have been shown to markedly impair manual dexterity and tactile sensitivity, increasing the risk of performance degradation and accidental injury in cold environments [22, 23]. Subjects felt very cold in their hands during the 60-min walking with wind (Fig. 5). Although fingers and toes exhibited the lowest skin temperature, their contribution to mean skin temperature was limited due to their smaller weighting. Thus, the decline in mean skin temperature was mainly driven by the cooling of the large frontal body areas exposed to the wind, while the pronounced distal cooling accentuated local cold discomfort.

### Overall and peripheral thermal sensation

Moderate exercise could generate sufficient heat to compensate for heat loss in cold conditions [24], but the present results suggest that the everyday winter clothing is not sufficient to support comfort in strong wind conditions. Localized cold discomfort was noted, particularly in the extremities (hands and feet), which points to the need for better insulation for these areas. Notably, the overall thermal sensation exhibited patterns similar to those of the hands and feet, suggesting that thermal perception was more strongly influenced by peripheral skin temperature than by deep body temperature [25, 26]. Under conditions where peripheral regions are significantly cooled, the pronounced cooling of extremities likely dominated the afferent thermal signals, causing the whole-body thermal sensation to reflect peripheral rather than central thermal status [27].

In the present study, the WCT ranged from −5 to –12 °C, and the CSI scores were below 2, remaining within the “mild cold” category. The CSI scores suggest that the thermal burden in everyday winter conditions with proper clothing is manageable for most people. However, improving the insulation of gloves and footwear is necessary when facing strong winds, even in the mild cold category. These findings support the view that overall thermal sensation under windy cold conditions is dominated by peripheral afferent inputs rather than core temperature regulation, highlighting the need for thermal protection focused on distal extremities.

### What is an efficient way to measure everyday cold stress?

In the present study, the first half of the CSI formula became zero because the rectal temperature gradually increased during cold exposure. In this case, the CSI is a function of mean skin temperature (the last half of the CSI formula). As we found strong correlations between thermal sensations, thermal comfort, hand dorsum temperature, and the CSI, cold stress from strong winds in mild cold temperatures could be estimated using noninvasive measures, such as peripheral skin temperatures and subjective responses. In particular, hand dorsum temperature was a reliable reflection of cold exposure, especially in windy conditions. These findings align with previous studies that used skin temperature as a proxy for cold strain in real-world conditions [28]. That is, given that core temperature remained stable, monitoring hand temperature, thermal sensation, and comfort are sufficient to assess cold stress in everyday situations.

### What are the efficient methods for maintaining thermal comfort?

The present study showed that wind significantly reduced the thermal insulation of the clothing ensemble, and the clothing insulation at a wind speed of 4.5 m·s^−1^ was reduced down to 55% (2.04 clo, 1.42 clo, 1.12 clo, and 0.80 clo, respectively). Winslow and his colleague reported that clothing insulation decreases with air movement, and that windproof designs are crucial in maintaining thermal comfort [29]. Under windy cold conditions, the decrease in clothing microclimate temperature was significant only at the highest air velocity (7 m·s^−1^). This limited response likely reflects the low air permeability and multilayered structure of the winter padded jacket, which restricted convective disturbances from penetrating into the innermost air layer [30, 31]. In contrast, the clothing microclimate humidity decreased notably even at moderate wind speeds (≥ 2 m·s^−1^), suggesting that vapor diffusion and the movement-induced bellows effect facilitated moisture removal. These findings indicate that wind and body movement mainly affected humidity removal rather than heat exchange under mild cold conditions.

No visible sweating was observed during the 60-min walking, and total body mass loss did not differ among wind conditions, indicating that sweating remained minimal. The considerable body water loss (186–200 g over 80 min) reflects increased insensible water loss from both respiratory and cutaneous routes during mild exercise in cold conditions. This interpretation is consistent with previous findings showing that under mild cold exposure, respiratory water loss increases substantially due to the humidification of cold, dry inspired air, and sweat secretion is suppressed [32, 33].

Therefore, wind-resistant outer layers and filling materials are essential to preventing cold discomfort and potential injuries. In particular, still air within the filling materials should be preserved when exposed to strong winds. Air-padding jackets could be designed for this purpose [34]. Despite subjects wearing both gloves and shoes, finger temperatures were on average 5 °C lower than toe temperatures, likely due to the thicker insulation provided by shoes and socks compared to gloves.

### The role of body surface area in cold sensation and shivering response

The study found a significant negative correlation between body surface area (BSA) and overall thermal sensation and comfort, as well as hand thermal sensation and comfort, indicating that individuals with larger BSA perceived stronger cold stress. BSA was also significantly negatively correlated with toe temperature (ρ = −0.585, *P* = 0.001), suggesting that individuals with larger surface areas experienced greater peripheral heat loss, particularly in the distal extremities. These findings indicate that individuals with a larger BSA perceive cold conditions more acutely, as larger surface areas are more sensitive to cold environments due to increased heat loss from greater surface area [37]. On the other hand, BSA per unit weight showed a significant negative correlation only with overall thermal comfort. Because BSA per unit weight reflects the balance between heat loss and metabolic heat production, its effect may be limited under mild cold conditions, where changes in metabolic rate are less pronounced [38]. In this study, the mild cold stimuli and relatively small differences in body weight among the subjects likely reduced the prominence of the effect of BSA per unit weight. Conversely, when core temperature remained stable, the impact of BSA differences on heat loss appeared more pronounced, resulting in a clearer correlation with thermal sensation [39].

In the present study, shivering was more pronounced in the peripheral regions, such as the hands and feet, rather than in the trunk. This can be attributed to peripheral vasoconstriction, which prioritizes the maintenance of core temperature by reducing skin blood flow and accelerating distal cooling. Consequently, the peripheral muscles reached the shivering threshold earlier than trunk muscles. Furthermore, across wind conditions, BSA was positively correlated with total shivering frequency (ρ = 0.534, *P* = 0.002), reinforcing that individuals with larger surface areas tended to shiver more often, consistent with their greater sensitivity to cold sensation. The enhanced convective heat loss accompanying larger surface area likely promotes more sustained activation of shivering thermogenesis. Collectively, these findings highlight that morphological factors—particularly BSA—play a more critical role than BMI in determining peripheral thermal responses and vasoconstriction under mild cold stress [26, 35, 36]. From an anthropological perspective, this is in line with Allen’s and Bergmann’s rules, which describe how body shape and size adapt to environmental temperatures: larger surface areas relative to body mass enhance heat exchange and cold perception, especially under convective stress [40–42]. Thus, evaluating BSA provides a more comprehensive understanding of individual cold responses and offers practical insight for designing cold-protective clothing that accommodates morphological diversity.

## Conclusions

This study examined the physiological and psychological responses of young males while wearing typical winter clothing (*I*_T_, 2.1 clo) and walking for 60 min in −5 °C with four wind conditions (0 m·s^−1^, 2 m·s^−1^, 4.5 m·s^−1^, 7 m·s^−1^). The wind chill temperature under the present experimental conditions ranged from −5 to 12 °C, which is categorized as mild cold. We found that rectal and gastrointestinal temperatures were not reduced, and metabolic rate did not increase even while facing the strong wind (−7 m/s), while peripheral skin temperatures (hand, finger, foot, and toe) and psychological responses were significantly affected by the strong wind. These results suggest that the selected winter clothing provides adequate insulation for maintaining core body temperature but is not sufficient to support peripheral insulation and thermal comfort. Noninvasive indicators, including hand temperature, thermal sensation, thermal comfort, and wind speed sensation, could predict the physiological impacts of wind exposure up to 7 m·s^−1^ in mild cold temperatures. Further, we found that body mass index (BMI) showed no significant association with shivering frequency, whereas body surface area (BSA) was significantly and positively correlated with total shivering frequency. This finding might indicate that individuals with larger surface areas experienced stronger cold sensitivity, but this interpretation should be made with caution because the morphological range of the subjects was limited (BMI 21.6~27.2, BSA 1.75~2.14 m^2^). This study provides valuable insights for designing winter clothing and ensuring safe outdoor activities in mild cold environments, emphasizing the importance of protecting extremities and considering morphological diversity from wind chill.

## Data Availability

The datasets used and/or analyzed during the current study are available from the corresponding author upon reasonable request.

## Abbreviations

CSI: Cold Strain Index
*T*_re_: Rectal temperature
*T*_pill_: Gastrointestinal temperature
*T*_ac_: Auditory canal temperature
BMI: Body mass index
BSA: Body surface area
